# Altered expression of miR-152 and miR-148a in ovarian cancer is related to cell proliferation

**DOI:** 10.3892/or.2022.8285

**Published:** 2022-02-15

**Authors:** Xin Zhou, Fang Zhao, Zhen-Ning Wang, Yong-Xi Song, Hua Chang, Yeunpo Chiang, Hui-Mian Xu

Oncol Rep 27: 447-454, 2012; DOI: 10.3892/or.2011.1482

Following the publication of the above paper, an interested reader drew to the authors' attention that, in Fig. 5D, the data panels selected to represent the ‘SKOV3 with miR-148a mimics’ and ‘SKOV3 with Negative Control’ experiments appeared to contain overlapping data, such that they may have been derived from the same original source.

The authors have re-examined their original data, and realized how the errors in the compilation of Fig. 5 arose. The corrected version of Fig. 5, showing the correct data for the ‘SKOV3 with miR-148a mimics’ panel in Fig. 5D and the ‘SKOV3 with Negative Control’ panel in Fig. 5C, is shown on the next page. Note that these errors did not affect the overall conclusions reported in the study. The authors are grateful to the Editor of *Oncology Reports* for allowing them the opportunity to publish this Corrigendum; furthermore, they apologize for any inconvenience caused to the readership of the Journal.

## Figures and Tables

**Figure 5.**
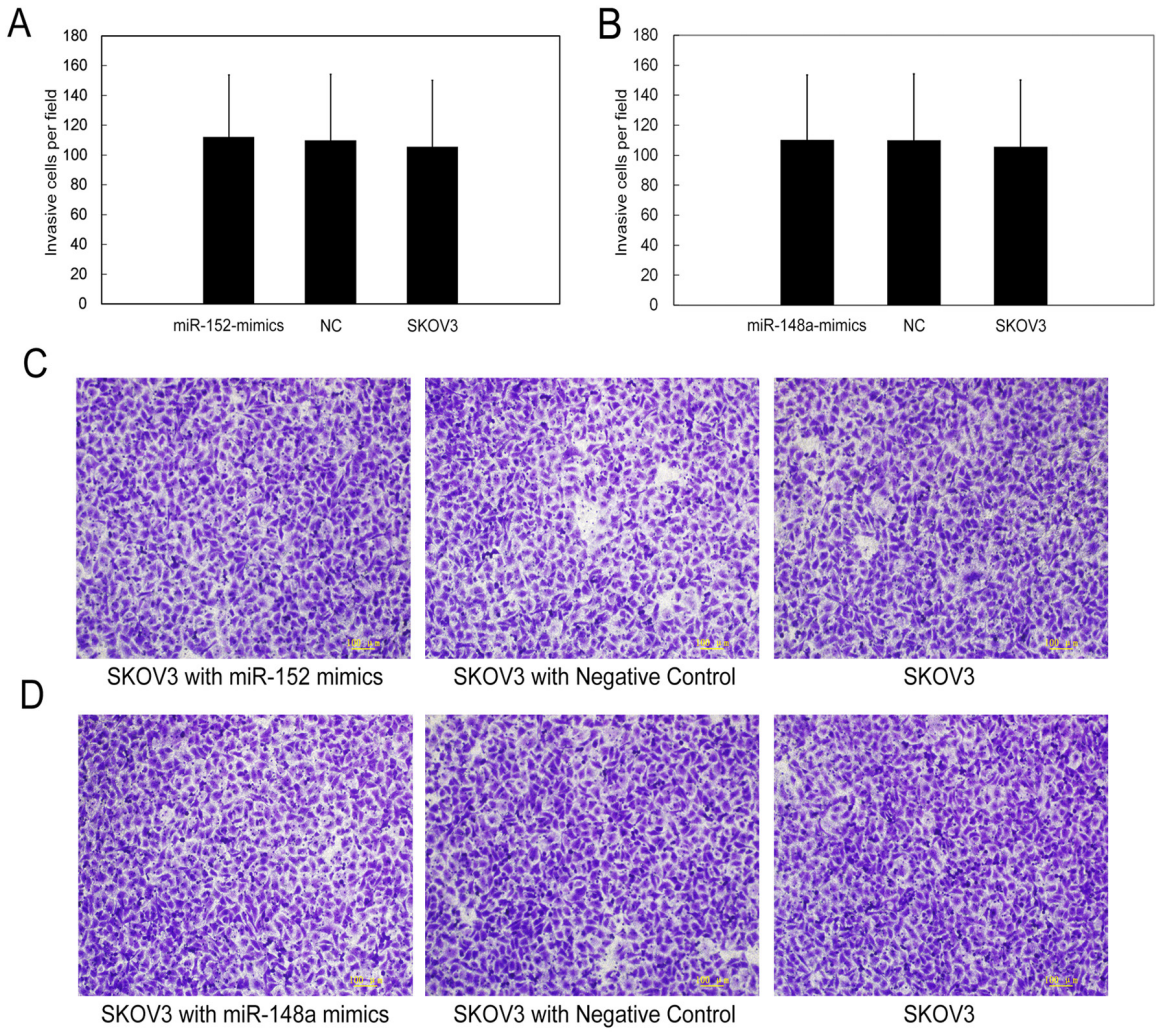
miR-152 and miR-148a do not inhibit cell invasion. The Transwell asssy was used to detect the invasion of SKOV3 cells. There were no significant differences between SKOV3 cells transfected with miR-152 or miR-148a mimics and negative control (NC). (A and B) Cell numbers traversing the membrane were determined by randomly counting in nine random fields under a microscope at a magnification of ×200. The bars represent the mean ± SD of three independent experiments. (C and D) Magnification, ×100.

